# Diet induced the change of mtDNA copy number and metabolism in Angus cattle

**DOI:** 10.1186/s40104-020-00482-x

**Published:** 2020-07-21

**Authors:** Ying Bai, José A. Carrillo, Yaokun Li, Yanghua He, Jiuzhou Song

**Affiliations:** 1 College of Life Sciences and Food Engineering, Hebei University of Engineering, Handan, 056038 China; 2 Department of Animal & Avian Sciences, University of Maryland, College Park, MD 20742 USA; 3 Council on Dairy Cattle Breeding, Bowie, MD 20716 USA; 4 Human Nutrition, Food and Animal Sciences, University of Hawaii at Manoa, Honolulu, HI 96822 USA

**Keywords:** Beef quality, Different diet, Gene expression, Metabolism, MtDNA

## Abstract

**Background:**

Grass-fed and grain-fed Angus cattle differ in the diet regimes. However, the intricate mechanisms of different beef quality and other phenotypes induced by diet differences are still unclear. Diet affects mitochondrial function and dynamic behavior in response to changes in energy demand and supply. In this study, we examined the mtDNA copy number, mitochondria-related genes expression, and metabolic biomarkers in grass-fed and grain-fed Angus cattle.

**Results:**

We found that the grass-fed group had a higher mtDNA copy number than the grain-fed group. Among different tissues, the mtDNA copy number was the highest in the liver than muscle, rumen, and spleen. Based on the transcriptome of the four tissues, a lower expression of mtDNA-encoded genes in the grass-fed group compared to the grain-fed group was discovered. For the mitochondria-related nuclear genes, however, most of them were significantly down-regulated in the muscle of the grass-fed group and up-regulated in the other three tissues. In which, *COX6A2*, *POLG2*, *PPIF*, DCN, and *NDUFA12*, involving in ATP synthesis, mitochondrial replication, transcription, and maintenance, might contribute to the alterations of mtDNA copy number and gene expression. Meanwhile, 40 and 23 metabolic biomarkers were identified in the blood and muscle of the grain-fed group compared to a grass-fed group, respectively. Integrated analysis of the altered metabolites and gene expression revealed the high expression level of *MDH1* in the grain-fed group might contribute to the mitochondrial NADH oxidation and spermidine metabolism for adapting the deletion mtDNA copy number.

**Conclusions:**

Overall, the study may provide further deep insight into the adaptive and regulatory modulations of the mitochondrial function in response to different feeding systems in Angus cattle.

## Background

Recently, grass-fed beef production is booming in the United States, which is considered more beneficial for human health [1]. The main difference between grass-fed and grain-fed beef cattle is attributable to the diet regimes. As we know, mitochondria play an essential role in the cellular response to environmental stressors, such as air, water, temperature, and food [2]. Mitochondria are dynamic organelles that are present in almost eukaryotic cells and play a crucial role in several cellular pathways, producing most of the cellular ATP employing oxidative phosphorylation (OXPHOS). Different tissues have specialized mitochondrial features due to differences in their metabolic profiles and energy demands [3–6]. The difference is not only restricted to the OXPHOS function [6] and mitochondria protein compositions [7] but also gene expressions, mitochondrial DNA maintenance, and replications [8, 9].

Mitochondria contain their genomes (mtDNA), encoding a total of 13 proteins, together with 22 tRNAs and two rRNAs, necessary for translations of the respiratory subunit mRNAs within the mitochondrial matrix. Alterations of mtDNA mean inactivating genetic mutations or depletion of mtDNA copy numbers [10]. The copies of mtDNA serve as the initial template for mtDNA replication and maintenance, allowing cells to acquire the appropriate numbers of mtDNA copy as they differentiate into mature cell types [11–13]. Mitochondria replication, maintenance as well as gene expression are controlled by nuclear-encoded factors, such as nuclear respiratory factor 1 and 2 (*NRF-1*, *NRF-2*), mitochondrial transcription factor A (*TFAM*), and peroxisome proliferator-activated receptor-gamma coactivator-1a (*PGC-1a*) [14, 15]. The mitochondrial and nuclear genomes coordinate and co-evolve in eukaryotes to adapt to environmental changes. Variation in the mitochondrial genome is capable of affecting the expression of genes on the nuclear genome [16]. However, how the diet regime influences the mitochondria dynamics, further associated with beef quality, has yet to know.

Provisioning of dietary macronutrients to mitochondria is influenced by genetic variations that affect the activities of the electron transport system, retrograde organelle signaling to the nuclear genome, and anterograde signaling to the mitochondrion [17, 18]. In diet-induced obesity, impaired mitochondrial function and decreased mitochondria contents were found in liver and skeletal muscle [19, 20]. Dietary supplementation with plasminogen increased mitochondrial copy number and improved mitochondrial function in mice [19–21]. A reduction of the mtDNA copy number and the expression of genes involved in mitochondrial biogenesis in the liver of rats fed a high-fructose diet [22]. When feeding flies with a high carbohydrate diet, the mitochondria-related gene expression, and mtDNA were changed [23]. RNA-seq analysis revealed that genes associated with mitochondrial function were differentially expressed between low-protein fed heifers on high- or low- energy diets [24].

Meanwhile, diet by mitochondrial DNA haplotype interactions drives metabolic flexibility and organismal fitness [23]. Metabolomic profiling provides an additional layer of knowledge for the complete representation of the phenotype of the animal, revealing the combined contributions of gene expression, enzyme activity, and environmental context [23, 25]. What a cow eats can have a significant effect on the metabolic and nutrient composition of the beef via modulating metabolism and energy expenditure [1]. Grass-fed beef may contain less total fat than grain-fed beef, but a lot more omega-3 fatty acids and conjugated linoleic acid [26–28]. In our previous studies, the alterations in divergences in free fatty acids, lipid levels, and gene expression patterns have been observed between grass-fed and grain-fed Angus cattle [29–31]. However, information on the possible mechanism on mitochondrial function and metabolism between the grass-fed and grain-fed is lacking. Here, we propose using trio measurements of mtDNA copy number, mRNA transcribed from mtDNA, and mRNA transcribed from nuclear DNA- related to mitochondria, to explore mitochondrial roles responded to different diets in Angus cattle. In parallel, we combined these data with metabolomics to assess the correlation between mitochondria-related genes and metabolites levels. These findings additionally provide insights into patterns of transcriptional coordination between the mitochondrial and nuclear genomes.

## Methods

### Animals and ethics statement

The grass-fed and grain-fed Angus cattle were raised at the Wye Angus farm, which has been closed more than 70 years, have similar genetics. The grain-fed group received a conventional diet comprised of shelled corn, corn silage, soybean, and trace minerals. The grass-fed steers usually consumed grazed alfalfa. The diet composes, and feeding regiments for grass-fed and grain-fed cattle are described in Additional file 1: Table S1. The longissimus dorsi muscle, liver, spleen, and rumen tissues were collected from steers with grass-fed and grain-fed, respectively, when they reached the market weight. The grain-fed animals reached the market weight around the age of 14 months, while grass-fed steers needed approximately 200 additional days to reach a similar weight value. Samples were taken immediately after euthanasia, frozen in liquid nitrogen, and stored at − 80 °C until used for extracting RNA and DNA. Blood collection for both groups was performed before slaughtering. The metabolomics profiling analysis were performed in blood and muscle samples from eight individuals of the grass-fed group and the grain-fed group, respectively. Then, the four tissues from two randomly selected individuals in each group were used for deep sequencing. MtDNA copy number was detected in 24 samples (4 tissues × 3 individuals × 2 groups).

All animal experiments were conducted according to the NIH guidelines for housing and care of laboratory animals and following the regulations of the University of Maryland at College Park (UMCP). The UMCP Institutional Animal Care and Use Committee (IACUC) reviewed and approved the protocols (permit number R-08-62).

### mtDNA copy number analysis

DNA was prepared, and mtDNA copy number analysis was performed according to the previous report [32, 33]. Briefly, total DNA was extracted from longissimus dorsi muscle, liver, spleen, and rumen tissues using Wizard Genomic DNA Purification Kit (Promega, Madison, WI, USA), respectively. The DNA concentration was detected using Nanodrop-2000 spectrophotometer (Thermo Fisher Scientific Inc., Wilmington, DE) and adjusted to 50 ng/μL. Primers were designed for four mitochondrial genes: the *ND2* (NADH dehydrogenase subunit 2), *ND5* (NADH dehydrogenase subunit 5), *CYTB* (cytochrome B), *12sRNA*, and *COX3* (cytochrome oxidase subunit III). The *ACTB* (actin B) gene was used as a nuclear control gene. For the analysis of mtDNA copy number, quantitative real-time PCR (qPCR) amplification of genomic DNA was performed on a C1000 Touch thermal cycler (BioRad, Hercules, CA, USA). The reactions for each gene were performed with 50 ng genomic DNA in triplicates in a final volume of 10 μL using 300 nmol/L of the specific primers and the 2 × SYBR Green PCR mix (Biorad, Hercules, CA, USA). The PCR program was 95 °C for 5 min, 40 cycles of 95 °C for 15 s, 60 °C for the 30 s, and 72 °C for 30 s, with a melting curve analysis (65 °C–95 °C) in the last cycle to evaluate amplification specificity. For each run, a standard curve was generated from 10-fold serial dilutions (10^− 1^ to 10^− 8^). Relative mtDNA copy numbers were calculated following equation [34]: MtDNA copy number = 2^1 + (Ct^_n_gene_^-Ct^_mt_gene_^)^, where Ct represents the average cycle threshold. The mtDNA copy number data from the four tissues were analyzed separately.

### RNA sequencing and analysis

The RNA extraction, cDNA synthesis, library preparation, transcriptome sequencing, and raw data were carried out from our reported protocols [29–31]. We employed FastQC v.0.11.2 (https://www.bioinformatics.babraham.ac.uk/projects/fastqc/) to check the sequence data quality and Trim Galore v 0.4.0 (https://www.bioinformatics.babraham.ac.uk/projects/trim_galore/) to clean the data. The cleaned data were aligned to the reference genome downloaded from Ensembl (Bos_taurus.ARS-UCD 1.2) using Hisat2 (https://ccb.jhu.edu/software/hisat2/) [35]. Differentially expressed genes were computed by using Cuffdiff (cole-trapnell-lab.github.io/cufflinks/cuffdiff). A gene was considered to be differentially expressed when fold changes ≥2 and *FDR* < 0.15. Gene Ontology (GO) enrichment analysis and Kyoto Encyclopedia of Gene and Genomes (KEGG) pathway analysis were implemented with STRING tools (http://string-db.org/) and KOBAS 3.0 (http://kobas.cbi.pku.edu.cn/).

### Metabolomics profiling

Metabolomics profiling analysis was performed by the Metabolon Platform, as previously described [29]. The whole blood and muscle tissues were assigned a unique identifier by the Metabolon Laboratory Information Management System (LIMS). Samples were prepared using the automated MicroLab STAR system from Hamilton Company. A recovery standard was added before the first step in the extraction process for quality control (QC) purposes. Sample preparation was conducted using aqueous methanol extraction process to remove the protein fraction while allowing for maximum recovery of small molecules. The resulting extract was divided into four fractions: one for analysis by liquid chromatography/mass spectroscopy (LC/MS) (positive mode), one for LC/MS (negative mode), one for gas chromatography/mass spectroscopy (GC/MS), and one for backup. Samples were placed briefly on a TurboVap (Zymark) to remove the organic solvent. Each sample was then frozen and dried under a vacuum. Samples were then prepared for the appropriate instrument. The progress of LC/MS, GC/MS, QA/QC, data extraction, and compound identification were detail described in our lab previous publication. The MUVR package in R was used to find the metabolic biomarkers [36].

### Metabolomic enrichment and pathway characterization

The pathway analysis was performed by MetaboAnalyst 4.0 [37]. The common pathway databases in the MetaboAnalyst 4.0 were used to explore metabolic impact pathways. The pathway with a *P*-value less than 0.05, as well as an impact value greater than 0.1, was defined as a significant impact pathway. Degree Centrality was selected for the node importance, and the pathway impact value calculation in the topological analysis. The hypergeometric distribution in the overrepresentation analysis was applied to assess the significance of each pathway based on its overlap with pathway lists in the joint databases. Statistical significance was defined at the joint *P* < 0.05.

## Results

### MtDNA copy number variation in different tissues

**Fig. 1:**
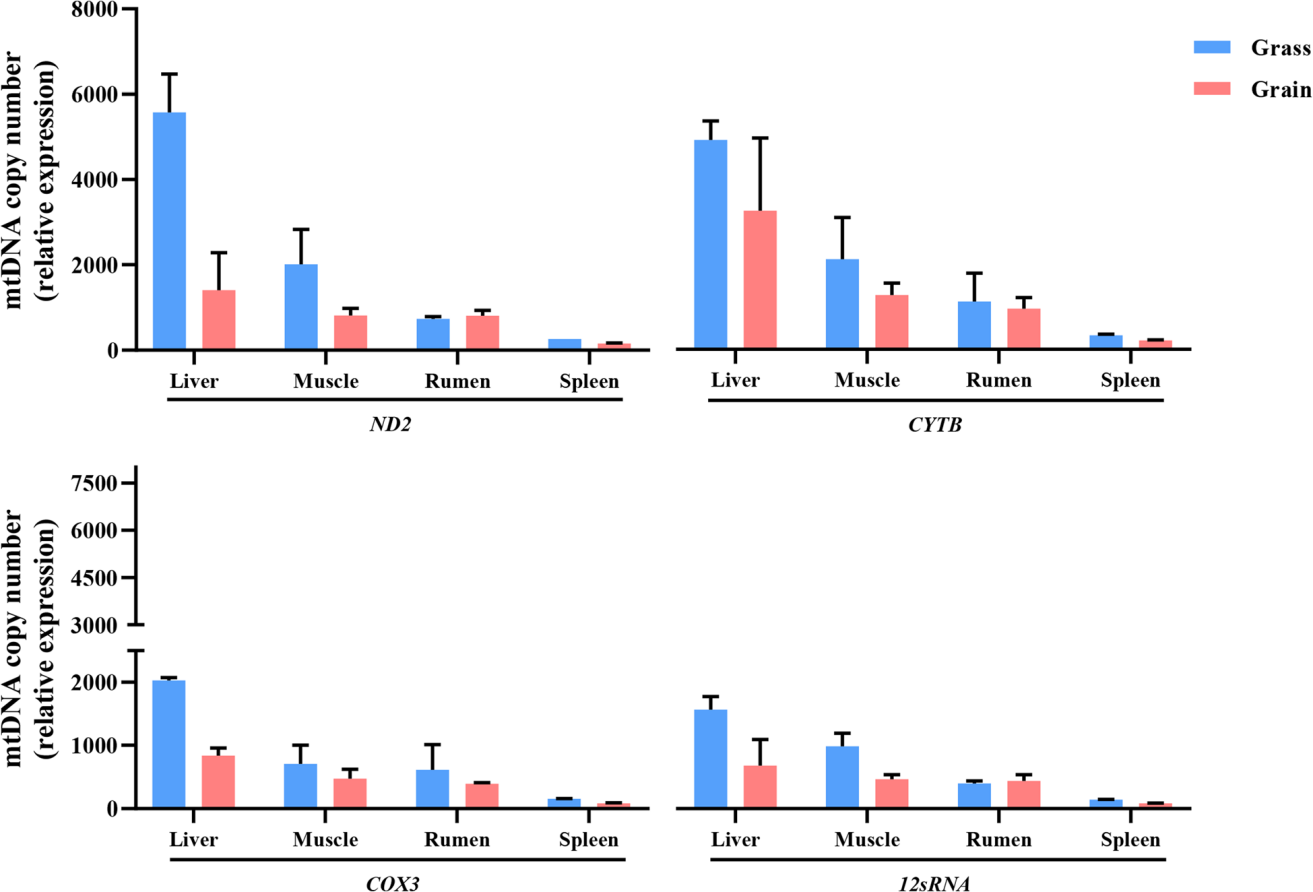
The mtDNA copy number variation in different tissues between grass-fed and grain-fed

MtDNA copy number, assayed with independent primer sets, showed the difference in grass-fed and grain-fed groups. Overall, grass-fed steers had higher mtDNA copy number than grain-fed steers. Among tissues, mtDNA copy number was higher in the liver than the other three tissues, regardless of grass-fed and grain-fed ones. The following was muscle. In regards to the *ND2/ACTB* primer set, diet significantly affected copy number. In liver and muscle, the mtDNA copy number showed significant differences between grass-fed and grain-fed (*P* < 0.05). But there was little influence on the copy number in rumen and spleen. Copy number with regards to *CYTB/ACTB*, *COX3/ACTB*, or *12SRNA/ACTB* showed a similar result (Fig. 1).

### The expression of mitochondria DNA-encoded genes

**Fig. 2:**
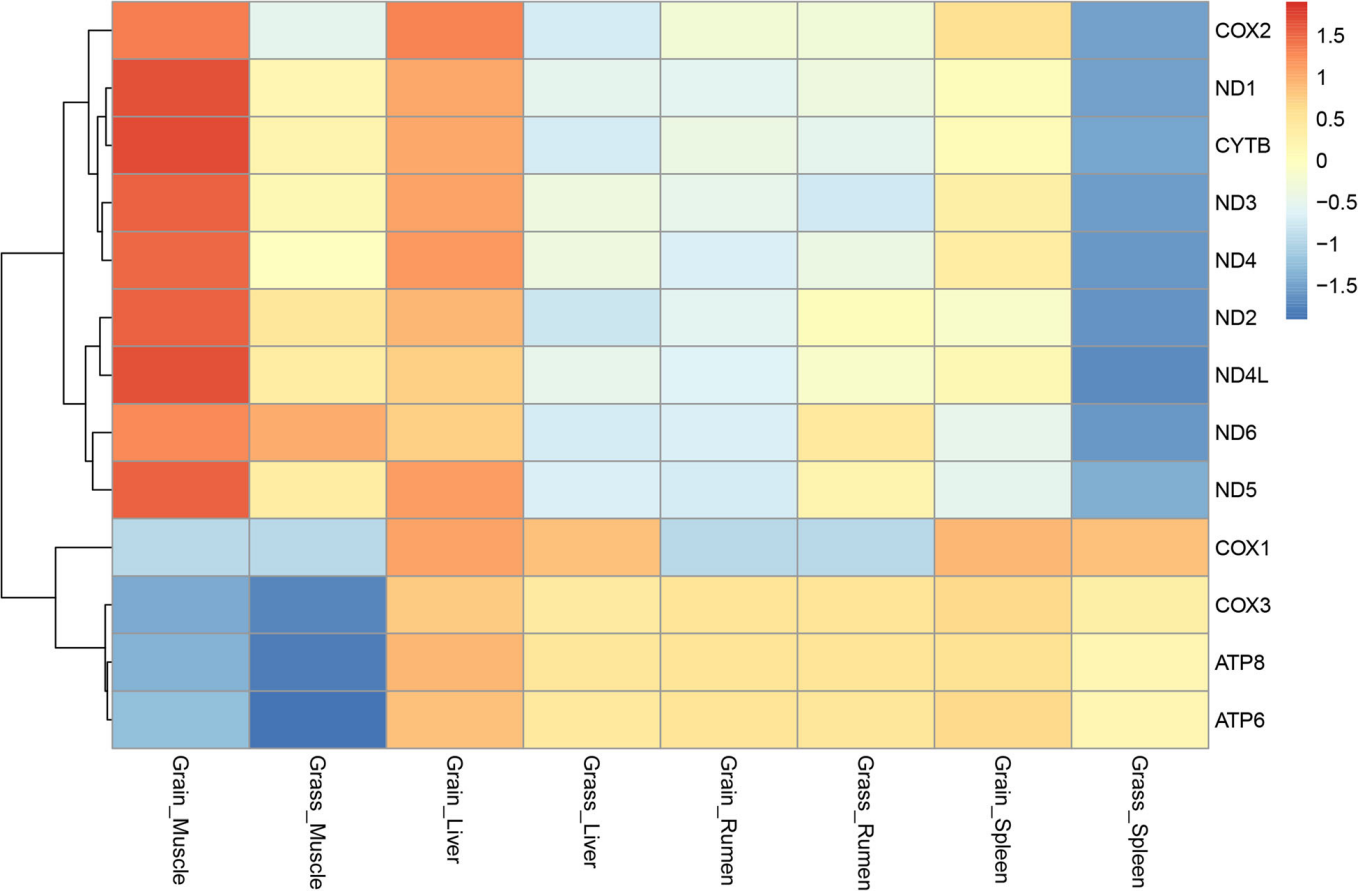
Heat map showing relative expression of mtDNA encoded genes in the four tissues of grass-fed and grain-fed Angus cattle. The average fragments per kilobase of transcript per million (FPKM) value of the two replicates was used as the gene expression, and the heat map was plotted based on log_2_(FPKM+ 1). The color legend represents the appropriate level, with red indicating high expression level and the blue indicating low expression level

To identify whether the mitochondrial gene expression was altered in grass-fed and grain-fed cattle, the 13 mtDNA-encoded genes were examined using RNA sequencing data (Fig. 2). In muscle, seven of the 13 genes, *ND1*, *ND2*, *ND3*, *ND4*, *ND5*, *COX2*, and *CYTB*, were significantly up-regulated in the grain-fed group compared to the grass-fed group. In the liver, *ND1*, *ND2*, *ND5*, *COX2*, and *CYTB* had lower expression levels in the grass-fed group. In the spleen, only one mtDNA-encoded gene, *ND3*, was showed significantly lower in the grass-fed group. In the rumen, there was no mtDNA-encoded gene was significantly expressed between the two diets. Interestingly, in the four tissues, no differentially expressed mtDNA-encoded genes were shown up-regulation in grass-fed Angus cattle.

### Differentially expressed mitochondria-related nuclear genes

The human mitochondria-related genes were downloaded from the MitoProteome Human Mitochondrial Protein Database [38]. Gene names were directly compared with those in cattle using the BioMart data-mining tool (http://useast.ensembl.org/biomart/martview). Meanwhile, the genes reported to be involved in mitochondrial function was generated by searching the “cattle mitochondrial” into the NCBI Gene database (National Center for Biotechnology Information, U.S. National Library of Medicine, Gene, http://www.ncbi.nlm.nih.gov/gene). Finally, 1283 genes were matched, including 13 mtDNA encoded protein genes as well as 1270 mitochondria-related nuclear genes (Additional file 1: Table S2).

**Fig. 3:**
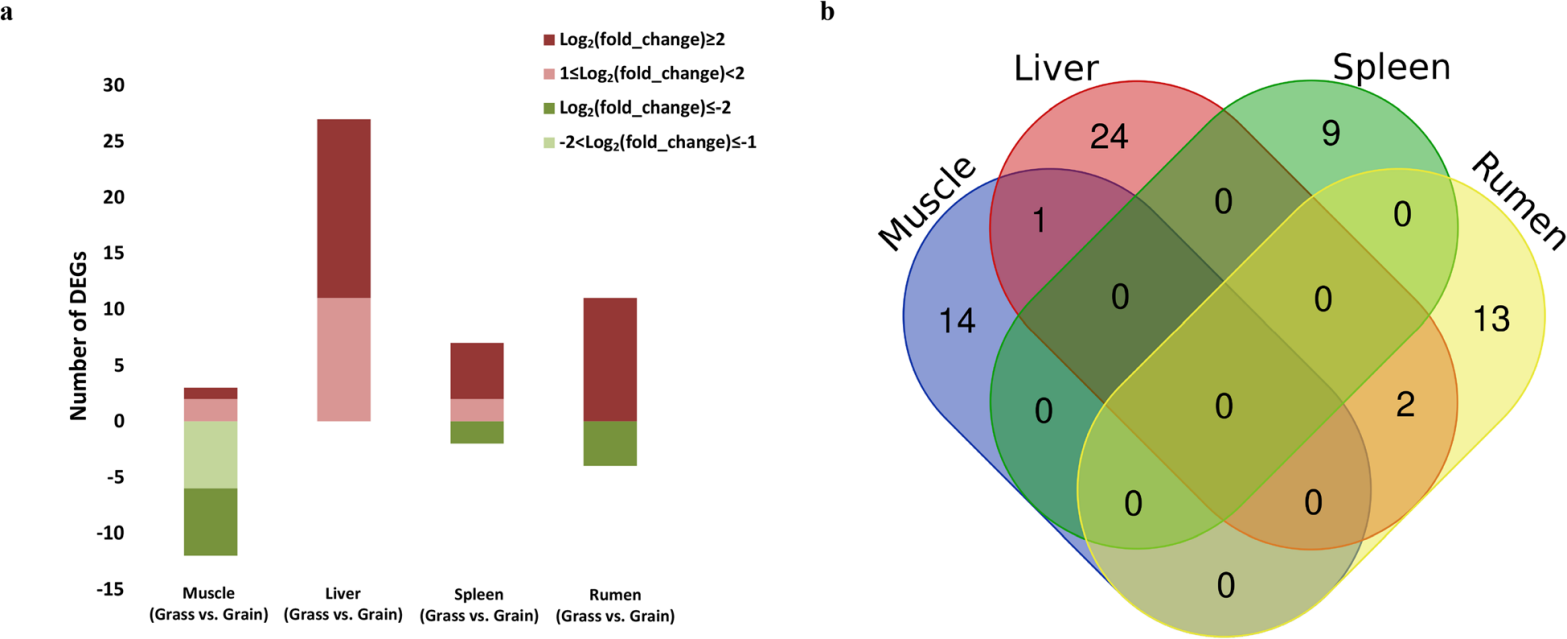
Differentially expressed mitochondria-related nuclear genes. **a** Number of up- or down-regulated DEGs in the four tissues. Up-regulated and down-regulated genes were displayed in red and green, respectively. **b** Venn diagram showing the number of DEGs commonly expressed in the four tissues

The expression levels of 1270 mitochondria-related nuclear genes were also investigated based on the RNA sequencing data, and differentially expressed gene (DEG) analyses were performed between the two diets across different tissues. The numbers of the differentially expressed that mitochondria-related nuclear genes were shown in Fig. 3 and Additional file 1: Table S3-S7. Compared to the grain-fed group, most of the DEGs were down-regulated in the muscle of the grass-fed group, but more DEGs were up-regulated in the other three tissues (Fig. 3a). In muscle, 15 mitochondria-related nuclear genes were differentially expressed in a diet-dependent manner. *CKMT2* (creatine kinase, mitochondrial 2), *UBB* (ubiquitin B), and *MDH1* (malate dehydrogenase 1) were up-regulated in the grass-fed group, while other nine genes had the opposite expression pattern. In the liver, the effect of diet change showed that 27 mitochondria-related nuclear genes were differentially expressed, all of which were up-regulated in the grass-fed group. Among the nine DEGs observed in spleen, seven genes were up-regulated, and two genes, *LYRM7* (LYR motif containing 7) and *CYP11A1* (cytochrome P450, family 11, subfamily A, polypeptide 1), were down-regulated in the grass-fed group. In the rumen, 15 mitochondria-related nuclear genes were differentially expressed (fold changes ≥2, *FDR* < 0.15), of which 11 genes were up-regulated, and four genes were down-regulated in the grass-fed compared to the grain-fed ones. There were no DEGs in common among the four tissues of the two different diets. *UBB* was commonly differentially expressed in muscle and liver. While there were two DEGs, *DHCR24* (24-dehydrocholesterol reductase) and *SHMT1* (serine hydroxymethyltransferase 1), commonly expressed in liver and rumen (Fig. 3b).

### Bioinformatics functions analysis

To further define the potential biological function of differentially expressed mitochondrial-related genes, including mtDNA encoded protein genes, as well as mitochondria-related nuclear genes, GO analysis and KEGG pathway analysis were performed. The enriched GO terms of biological processes, molecular functions and cellular components were shown in Table 1 and Additional file 1: Table S8 (*FDR* < 0.05). For the KEGG pathway analysis, most of the differentially expressed pathways (*P* < 0.05) were associated with metabolic pathways in the four tissues (Additional file 2: Fig.S1). In muscle, the metabolic pathways included carbon metabolism, glyoxylate and dicarboxylate metabolism, and butanoate metabolism. In liver, the metabolism were involved in pyruvate metabolism, amino acid metabolism, glyoxylate, and dicarboxylate metabolism, carbon metabolism, and glycerolipid metabolism. Meanwhile, The FoxO signaling pathway was also differentially expressed, which was associated with development.

**Table 1:** Top five gene ontology terms in the four tissues

Tissue	GO terms	FDR
Muscle	Biological Process	
	Cellular respiration	1.51E-11
	Oxidation-reduction process	4.18E-11
	ATP synthesis coupled electron transport	8.73E-10
	Nucleobase-containing small molecule metabolic process	5.99E-09
	ATP metabolic process	5.28E-08
	Cellular Component	
	Mitochondrion	8.58E-14
	Mitochondrial respiratory	6.93E-13
	Respiratory chain complex	6.93E-13
	An inner mitochondrial membrane protein complex	4.88E-12
	Mitochondrial part	1.80E-11
	Molecular Function	
	Oxidoreductase activity	2.07E-12
	NADH dehydrogenase (ubiquinone) activity	6.48E-09
	Catalytic activity	6.57E-09
	Electron transfer activity	0.00029
	Cytochrome-c oxidase activity	0.0025
Liver	Biological Process	
	Oxidation-reduction process	3.08E-17
	Small-molecule metabolic process	1.32E-13
	Metabolic process	9.41E-11
	Cellular metabolic process	9.41E-11
	Cellular process	1.04E-08
	Cellular Component	
	Mitochondrion	8.72E-21
	Mitochondrial part	6.00E-19
	Cytoplasmic part	1.95E-13
	Cytoplasm	4.00E-13
	Mitochondrial membrane	1.82E-11
	Molecular Function	
	Oxidoreductase activity	4.54E-16
	Catalytic activity	1.61E-12
	Cofactor binding	2.96E-06
	Aldehyde dehydrogenase (NAD) activity	2.75E-05
	Coenzyme binding	5.48E-05
Spleen	Biological Process	
	Oxidation-reduction process	0.0117
	Cellular Component	
	Mitochondrial part	7.57E-10
	Mitochondrial membrane	6.12E-09
	Mitochondrial inner membrane	4.01E-08
	Mitochondrial membrane part	6.04E-08
	Inner mitochondrial membrane protein complex	0.00012
	Molecular Function	
	Oxidoreductase activity	0.0014
	NADH dehydrogenase (ubiquinone) activity	0.003
	Catalytic activity	0.014
Rumen	Biological Process	
	Positive regulation of mitochondrion organization	0.0356
	Cellular metabolic process	0.0356
	Organonitrogen compound metabolic process	0.0356
	Cellular modified amino acid metabolic process	0.0419
	Phosphorylation	0.0419
	Cellular Component	
	Mitochondrial part	0.00044
	Mitochondrial envelope	0.0016
	Cytoplasm	0.0028
	Mitochondrial membrane	0.0098
	Cytoplasmic part	0.0126
	Molecular Function	
	Drug binding	0.00042
	Kinase activity	0.0046
	Catalytic activity	0.0076
	Transferase activity	0.0076
	Carbohydrate derivative binding	0.0076

### Prediction potential metabolic biomarkers and significant impact metabolic pathways

**Fig. 4:**
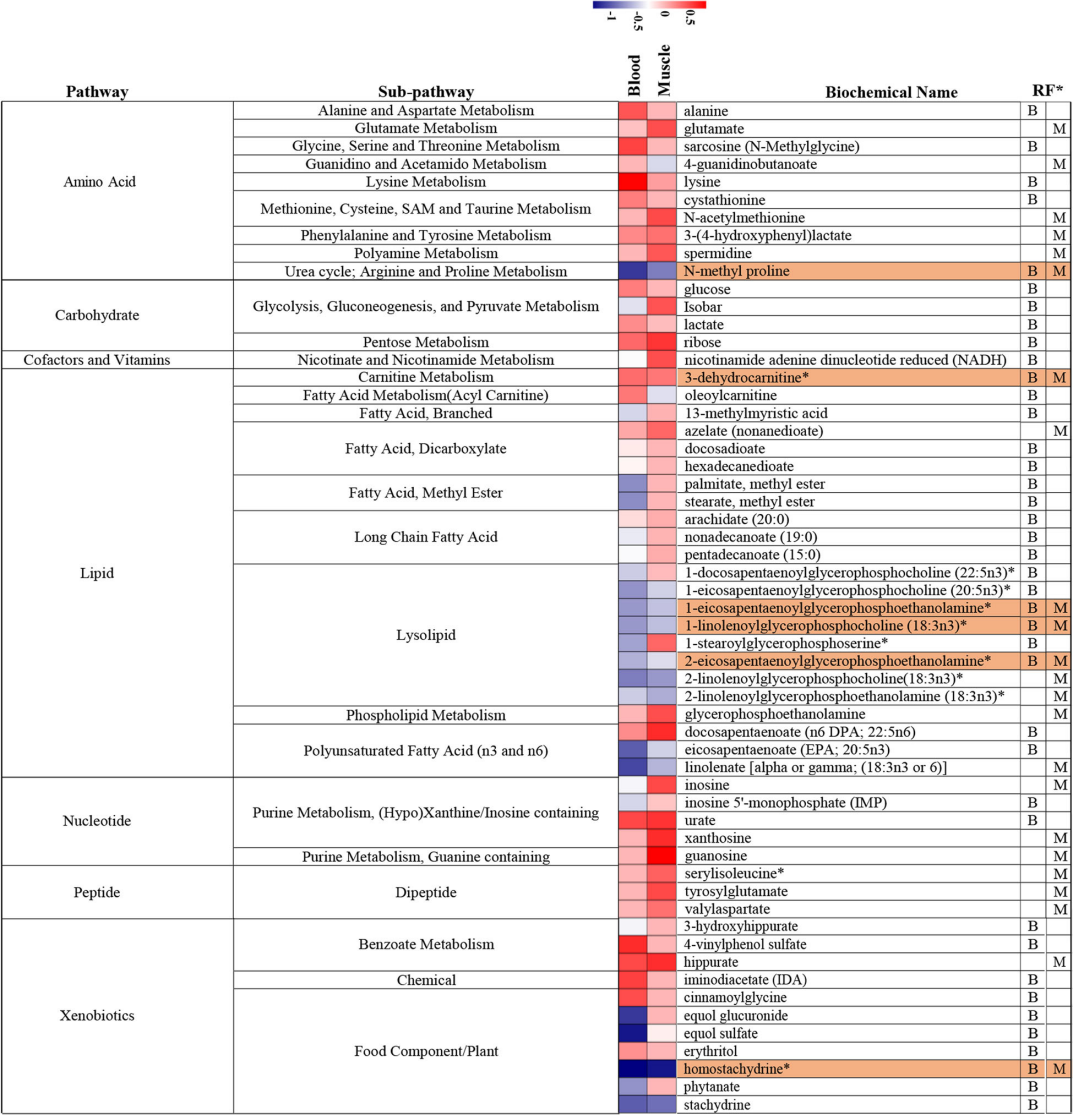
Potential metabolic biomarkers and metabolic pathways in blood and muscle of grain-fed group compared to the grass-fed group. B, Blood; M, Muscle; Color highlighting indicates the common six metabolites in the two tissues

LC/MS and GC/MS data detected 326 and 353 compounds in blood and muscle [29] (Additional file 3). Then, the compounds were analyzed using MUVR package in R to find themetabolic biomarkers. There were 40 and 23 metabolic biomarkers were identified in blood and muscle of the grain-fed group compared to a grass-fed group, respectively (Fig. 4). The involved metabolites belonged to the amino acid, carbohydrate, cofactors and vitamins, lipid, nucleotide, peptide, and xenobiotics. For the 40 metabolic biomarkers in blood, 14 metabolites had higher relative abundance in blood of the grain-fed group than the grass-fed group (*P* < 0.01). Another 26 metabolites significantly decreased in the grain-fed group. In muscle, 14 metabolic biomarkers were increased in a grain-fed group compared to the grass-fed group, and the other nine were decreased (*P* < 0.01). Among these metabolites, four lipids (1-eicosapentaenoyl glycerophosphoethanolamine, 2-eicosapentaenoyl glycerophosphoethanolamine, 1-linolenoyl glycerophosphocholine (18:3n-3), and 3-dehydrocarnitine), one amino acid (N-methyl proline), and one xenobiotic (homostachydrine) were proposed as conventional metabolic biomarkers for the two tissues, which had same abundance trend between the two different diet groups.

**Fig. 5:**
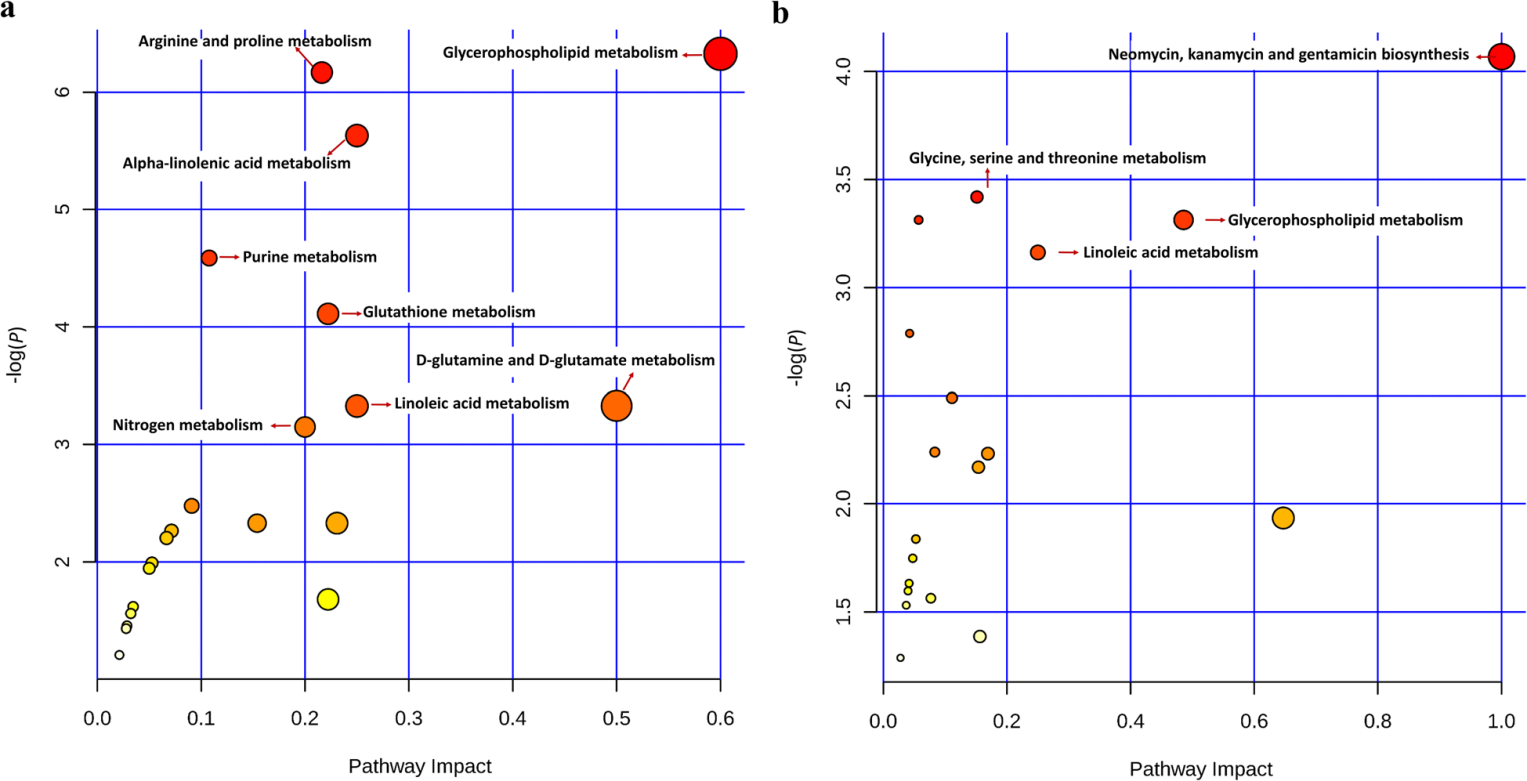
The pathway impact view of the metabolic biomarkers identified in muscle and blood tissue from the grass-fed and grain-fed group, respectively. **a** the pathway impact view of the metabolic biomarkers identified in muscle. **b** the pathway impact view of the metabolic biomarkers identified in blood. The X- and Y-axes represent the pathway impact value and pathway enrichment value, respectively; larger sizes and darker colors represent higher pathway enrichment and impact values

Impact pathway analysis revealed that eight pathways were significant impact pathways based on the 23 metabolic biomarkers in muscle (Fig. 5a): glycerophospholipid metabolism (*P* = 0.0018, impact value = 0.6), arginine and proline metabolism (*P* = 0.0021, impact value = 0.2162), alpha-linolenic acid metabolism (*P* = 0.0036, impact value = 0.25), purine metabolism (*P* = 0.0102, impact value = 0.1077), glutathione metabolism (*P* = 0.0164, impact value = 0.2222), linoleic acid metabolism (*P* = 0.0359, impact value = 0.25), D-glutamine and D-glutamate metabolism (*P* = 0.0359, impact value = 0.5), nitrogen metabolism (*P* = 0.0429, impact value = 0.2). Meanwhile, there were four significant impact pathways based the 40 metabolic biomarkers in blood (Fig. 5b): neomycin, kanamycin and gentamicin biosynthesis (*P* = 0.0171, impact value = 1), glycine, serine and threonine metabolism (*P* = 0.0328, impact value = 0.1515), glycerophospholipid metabolism (*P* = 0.0364, impact value = 0.4857), linoleic acid metabolism (*P* = 0.0423, impact value = 0.25).

### Integrated analysis of metabolites and mitochondrial-related genes

**Fig. 6:**
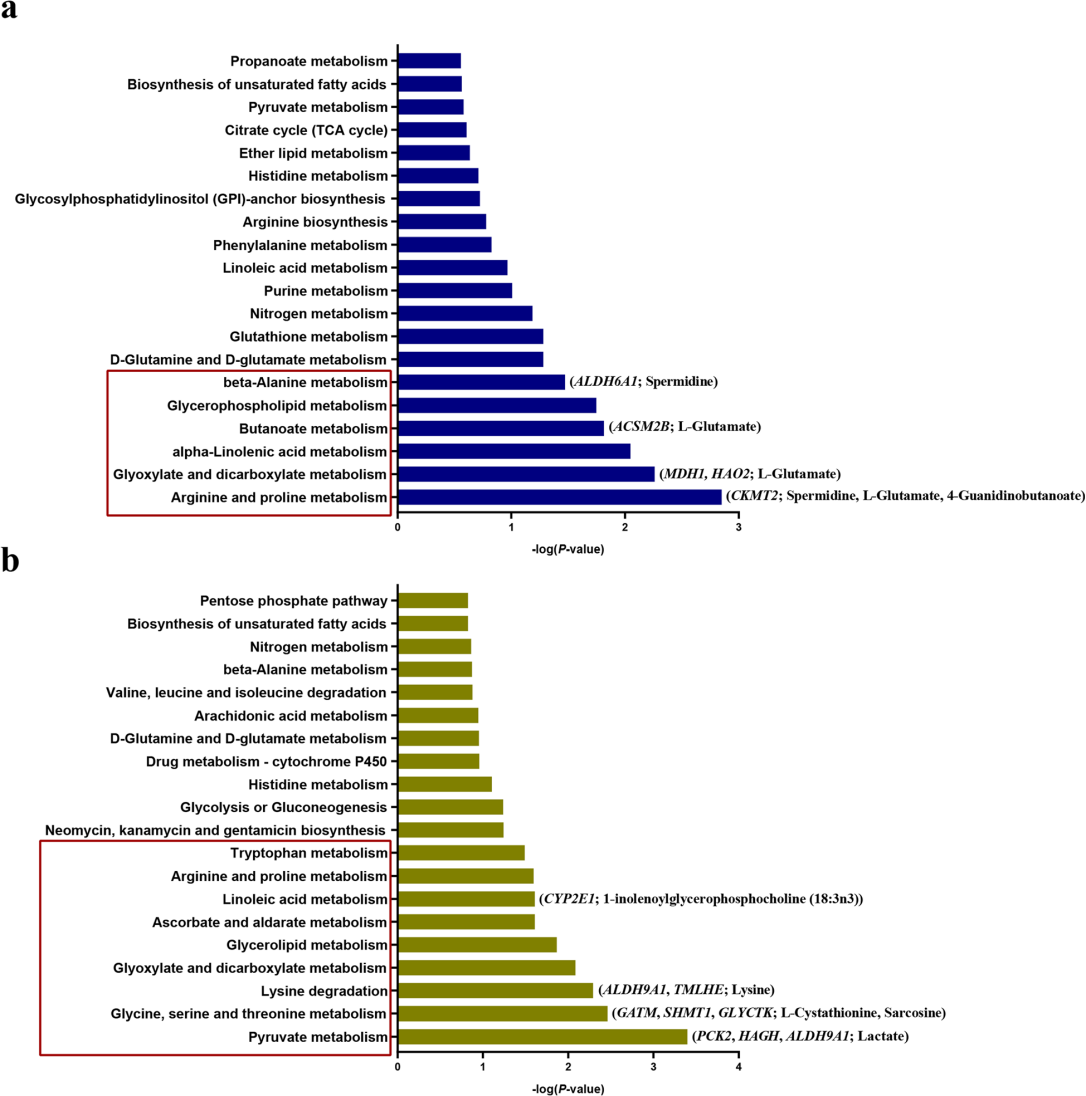
Integrated metabolic pathway analysis from combined metabolites and differentially expressed mitochondrial-related genes in (**a**) muscle or (**b**) blood. The red box represents the significantly enriched pathways

The over-representation analysis and pathway topology analysis were conducted between the diet-different metabolic biomarkers and differentially expressed mitochondrial-related genes in the muscle of grass-fed and grain-fed groups (analysis I). The nutrients digested in the rumen are absorbed by the rumen epithelial wall and are then transported to the mammary to the liver for glycogenesis, followed by transportation to other tissues through the bloodstream [39, 40]. Therefore, we integrated the differentially expressed mitochondria-related nuclear genes in the liver and rumen together to ascertain the metabolic biomarkers in the blood, and did the over-representation analysis and pathway topology analysis (analysis II). Topology analysis uses the structure of a given pathway to evaluate the relative importance of the genes/metabolites based on their relative locations. In the analysis I, 27 pathways were identified, and 20 of them were displayed in Fig. 6a. In analysis II, 46 pathways were identified, the top 20 of which were shown in Fig. 6b. Six and nine pathways were significantly enriched in the analysis I and analysis II, receptively. They include Arginine and proline, glyoxylate and dicarboxylate, alpha-Linolenic acid, butanoate, glycerophospholipid, and beta-Alanine metabolisms. Other metabolisms also include pyruvate, glycine, serine and threonine, lysine degradation, glyoxylate and dicarboxylate, and tryptophan metabolisms. The gene-metabolite interaction networks were also shown in Fig. 6.

## Discussion

### MtDNA copy number variation

According to the nutritional analysis of grass-diet and grain-diet, the contents of non-fiber carbohydrates and starch were much higher in the grain-diet, providing more total-digestible nutrients and available energy as well. Contrarily, proteins, lignin, fiber matter, and ash were more abundant in the grass- than grain-diet (Additional file 1: Table S1). It has been reported that when the flies were fed a high protein diet, the mtDNA copy number was increased compared to the high carbohydrate diet [23]. In our results, grass-fed steers had higher mtDNA copy number than grain-fed steers, which might be that the administration of insulin and amino acids enhances mitochondrial biogenesis and ATP production [41]. Meanwhile, the mtDNA copy number was higher in the liver than muscle, rumen, and spleen, regardless of grass- or grain-fed group. According to the ND2/ACTB primer set, the mtDNA copy number was 3.96-fold higher in the liver of the grass-fed group than the grain-fed group and was 2.46-fold higher in the muscle, which might be due to the different mechanism of mtDNA replication and maintenance in different tissues [9, 42]. The liver and kidney cells replicate their mtDNA using the asynchronous mechanism, skeletal muscle and brown fat with high OXPHOS activity employ a strand-coupled replication mode, combined with increased levels of recombination [9].

### The expression of mitochondria-related genes

By RNA-seq profiling, 13 mtDNA-encoded genes were diet-dependent and could be differently regulated at the level of transcription. Concerning differential expression, most of the genes encoding subunits of complex I (*ND1*, *ND2*, *ND3*, *ND4*, *ND5*), complex III (*CYTB*) and complex IV (*COX2*) were down-regulated in the grass-fed group, suggesting that the high protein diet might have an adverse effect on the mtDNA-encoded genes [10]. Mitochondrial Complexes I, III and IV are considered the energy-conserving core of the electron transport chain because they pump protons across the mitochondrial inner membrane, which is responsible for ATP production, and affecting mitochondrial function directly [43, 44]. Future studies should assay the ATP levels in the different tissues of the grass-fed group and grain-fed group. However, the higher expression levels of mtDNA-encoded genes were along with lower mtDNA copy number, implicating that compensation for differentially expressed mtDNA-encoded genes resulting from the different diet might be via transcriptional mechanisms, rather than changes to mtDNA ploidy. Several factors, including but not limited to mtDNA copy number, ultimately determine the steady-state abundance of mtRNAs and derived proteins in a cell [32, 33, 45], most of which were nuclear-encoded factors [16, 46].

To see if the mitochondrial related nuclear genome experience analogous changes in gene expression due to different diets, we examined the expression of mitochondria-related nuclear genes in different tissues of the grass-fed and grain-fed group. In muscle, an inspection of differentially expressed genes showed evidence for up-regulation of ATP synthesis coupled electron transport (*COX6A2*) in grain-fed steers. The COX6A2 protein is one subunit of the respiratory chain complex IV, of which expression is restricted to striated muscles [47, 48]. *COX6A2* has an important role in thermogenesis and whole-body energy metabolism and maybe a potential new target for therapy against high-fat diet-induced obesity or insulin resistance [49, 50]. In the liver, all of the differentially expressed genes were up-regulated in the grass-fed group. According to the GO term analysis, the *POLG2* gene was involved in the respiration electron transport chain. *POLG2* encodes the accessory subunits of DNA polymerase gamma, which is the only DNA replicative polymerase involved in the human mitochondria and is crucial for the replication and repair of mtDNA [51, 52]. *POLG2* could enhance interactions with the DNA template and increases both the catalytic activity. Mutations in *POLG2* have a dominant-negative effect and lead to multiple mtDNA deletions [53]. The higher level of *POLG2* might contribute to the larger amount mtDNA copy number in the liver of grass-fed steers compared to the grain-fed. In the rumen, *PPIF* was up-regulated, and *DCN* was down-regulated in the grass-fed group compared to the grain-fed group. These two genes were involved in the GO term of “positive regulation of mitochondrion organization”. PPIF is previously known as cypD, which is an important mitochondrial chaperone protein and well known for regulating mitochondrial function and coupling of the electron transport chain and ATP synthesis by controlling the mitochondrial permeability transition pore [54, 55].

Overexpression of *cypD* in HEK293 cell mitochondria increased respiratory activity, particularly the activity of complex III. This led to the increasing assembly of supercomplexes containing complexes I, III, and IV. At the same time, cypD binds to complex III and supercomplexes containing complex III and faster incorporation of complex III into these supercomplexes [56]. *DCN* is involved in multiple cellular functions such as proliferation, migration, and invasion and acts as a structural molecule, as well as a ligand for receptors [57, 58]. It has been reported that *DCN* potently attenuated mitochondrial respiratory complexes and mtDNA [59]. In the spleen, *NDUFA12* was involved in the GO term of “oxidation-reduction process”, which was up-regulated in the grass-fed group compared to the grain-fed group. *NDUFA12* was required for the formation of the extra membrane arm of mitochondrial complex I [60] and had the function of the stability of complex I [61]. Based on these results, it suggested that different mitochondria-related nuclear factors, involved in ATP synthesis, mitochondrial replication, transcription, and maintenance, might contribute to the mtDNA copy variation and changing of mtDNA expression in different tissues of grass-fed and grain-fed steers.

### Key metabolic pathways after single and integrated analysis

According to the previous study, alterations in glucose metabolism and divergences in free fatty acids were found between the grass-fed and grain-fed Angus steers [29]. In the present study, 40 and 23 metabolic biomarkers were identified in the blood and muscle of the grain-fed group compared to a grass-fed group, respectively. Based on the single metabolic pathway analysis, the most significantly impacted pathway in the muscle of grass-fed and grain-fed group was glycerophospholipid metabolism, which was also significantly different in the blood of the two different diet group. Changes in lipid abundance may also be indicative of altered membrane metabolism. Phosphoethanolamine, glycerophosphocholine, and glycerophosphoethanolamine were elevated in grain-fed muscle and may suggest increased membrane turnover, which may support enhanced tissue growth [62]. In blood, the most significantly impacted pathway was Neomycin, kanamycin, and gentamicin biosynthesis, which was also the top pointed in the combined level (enrichment and biological meaning). The involved metabolite was NADH, which was important for the TCA cycle. In contrast, NADH was elevated in the blood, but diminished elevated in the muscle of grass-fed compared to grain-fed cattle and may highlight differential energy regulation by the muscle versus other tissues in response to these diets. The related gene *MDH1* was down-regulated in the muscle of the grain-fed group compared to grass-fed, while up-regulated in the liver and rumen. MDH1 is a NAD(H)-dependent enzyme and a part of the malate–aspartate shuttle (MAS). The synthesis of cytosolic malate was via MDH1. MDH1 generates NAD+ upon the reduction of oxaloacetate to malate. MAS is important for intracellular NAD(H) redox homeostasis as it transfers, reducing equivalents across the mitochondrial membrane [63].

Meanwhile, the most critical pathway was arginine and proline metabolism based on the integrated analysis in muscle. Three metabolites (spermidine, *L*-glutamate, 4-guanidinobutanoate), and one gene (*CKMT2*) were involved in this pathway. *CKMT2* is a mitochondrial creatine kinase responsible for the transfer of high energy phosphate from the mitochondria to the cytosolic compartment, and at the same time for returning ADP to the mitochondrial respiratory system, thereby stimulating oxidative phosphorylation, which was negatively associated with spermidine, *L*-glutamate, and 4-guanidinobutanoate in our results. Spermidine, *L*-glutamate, and 4-guanidinobutanoate were elevated levels in grain-finished muscle and may reflect higher synthesis and availability for tissue growth. Spermidine displays pleiotropic effects that include anti-inflammatory properties, antioxidant functions, enhancement of mitochondrial metabolic function, and respiration [64]. ATP, as polyanion, is capable of suppressing the polycationic effect of spermidine [65]. However, the relationship between the *CKMT2* and spermidine needed to be further studied. Meanwhile, spermidine is a polyamine compound, and the higher level in grain-finished muscle might occur due to the maintenance of mitochondrial NADH oxidation that responded to lower mtDNA copy number [66].

## Conclusions

In summary, these data suggest that the grass and grain diets in cattle would cause the differences in the mtDNA copy number, mtDNA expression, and mRNA expression of selected nuclear genes involved in mitochondrial function. Meanwhile, the changes of mitochondria-related gene expression might contribute to the metabolites level variations. However, further research is required to better understand the relationships between mitochondria function, metabolic and molecular mechanisms, and beef quality.

## Supplementary information

**Additional file 1: Table S1.** Feed components analysis. **Table S2.** The 1283 mitochondria-related genes. **Table S3.** Differentially expressed mitochondria-related gene in the four tissues. **Table S4.** The differentially expressed genes in muscle of grass-fed and grain-fed Angus cattle. **Table S5.** The differentially expressed genes in liver of grass-fed and grain-fed Angus cattle. **Table S6.** The differentially expressed genes in rumen of grass-fed and grain-fed Angus cattle. **Table S7.** The differentially expressed genes in spleen of grass-fed and grain-fed Angus cattle. **Table S8.** Gene ontology terms in the four tissues.

**Additional file 2: Figure S1.** KEGG pathways differentially expressed between grass-fed and grain-fed Angus cattle. (A) muscle, (B) liver, (C) spleen, (D) rumen.

**Additional file 3: Table S1.** The 326 compounds were detected in blood. **Table S2.** The 353 compounds were detected in muscle.

## Data Availability

The datasets used and/or analysed during the current study are available from the corresponding author on reasonable request. The datasets analysed during the current study are available in the GEO DataSets repository. The web links to datasets were https://www.ncbi.nlm.nih.gov/geo/query/acc.cgi?acc=GSE70248, https://www.ncbi.nlm.nih.gov/geo/query/acc.cgi?acc=GSE67018, https://www.ncbi.nlm.nih.gov/geo/query/acc.cgi?acc=GSE63550, https://www.ncbi.nlm.nih.gov/geo/query/acc.cgi?acc=GSE145376

## Abbreviations

OXPHOS: Oxidative phosphorylation
mtDNA: Mitochondrial DNA
NRF: Nuclear respiratory factor
TFAM: Mitochondrial transcription factor A
PGC-1a: Peroxisome proliferator-activated receptor-gamma coactivator-1a
ND2: NADH dehydrogenase subunit 2
ND5: NADH dehydrogenase subunit 5
CYTB: Cytochrome B
COX3: Cytochrome oxidase subunit III
ACTB: Actin B
GO: Gene Ontology
KEGG: Kyoto Encyclopedia of Gene and Genomes
QC: Quality Control
LC/MS: Liquid Chromatography/Mass Spectroscopy
GC/MS: Gas Chromatography/Mass Spectroscopy
DEG: Differentially expressed gene
CKMT2: Creatine kinase, mitochondrial 2
UBB: Ubiquitin B
MDH1: Malate dehydrogenase 1
LYRM7: LYR motif containing 7
CYP11A1: Cytochrome P450, family 11, subfamily A, polypeptide 1
DHCR24: 24-dehydrocholesterol reductase
SHMT1: Serine hydroxymethyltransferase 1

## References

[1] Provenza FD, Kronberg SL, Gregorini P (2019). Is Grassfed meat and dairy better for human and environmental health?. Front Nutr..

[2] Manoli I, Alesci S, Blackman MR, Su YA, Rennert OM, Chrousos GP (2007). Mitochondria as key components of the stress response. Trends Endocrinol Metab..

[3] Jayashankar V, Mueller IA, Rafelski SM (2016). Shaping the multi-scale architecture of mitochondria. Curr Opin Cell Biol..

[4] Kunz WS (2003). Different metabolic properties of mitochondrial oxidative phosphorylation in different cell types--important implications for mitochondrial cytopathies. Exp Physiol..

[5] Johnson DT, Harris RA, Blair PV, Balaban RS (2007). Functional consequences of mitochondrial proteome heterogeneity. Am J Physiol Cell Physiol..

[6] Pohjoismaki JL, Goffart S (2017). The role of mitochondria in cardiac development and protection. Free Radic Biol Med..

[7] Johnson DT, Harris RA, French S, Blair PV, You J, Bemis KG (2007). Tissue heterogeneity of the mammalian mitochondrial proteome. Am J Physiol Cell Physiol..

[8] Pohjoismaki JL, Goffart S (2011). Of circles, forks and humanity: topological organisation and replication of mammalian mitochondrial DNA. Bioessays..

[9] Herbers E, Kekalainen NJ, Hangas A, Pohjoismaki JL, Goffart S (2019). Tissue specific differences in mitochondrial DNA maintenance and expression. Mitochondrion..

[10] El-Hattab AW, Scaglia F (2013). Mitochondrial DNA depletion syndromes: review and updates of genetic basis, manifestations, and therapeutic options. Neurotherapeutics..

[11] Lee W, Johnson J, Gough DJ, Donoghue J, Cagnone GL, Vaghjiani V (2015). Mitochondrial DNA copy number is regulated by DNA methylation and demethylation of POLGA in stem and cancer cells and their differentiated progeny. Cell Death Dis..

[12] Dickinson A, Yeung KY, Donoghue J, Baker MJ, Kelly RD, McKenzie M (2013). The regulation of mitochondrial DNA copy number in glioblastoma cells. Cell Death Differ..

[13] Facucho-Oliveira JM, St John JC (2009). The relationship between pluripotency and mitochondrial DNA proliferation during early embryo development and embryonic stem cell differentiation. Stem Cell Rev Rep..

[14] Virbasius JV, Scarpulla RC (1994). Activation of the human mitochondrial transcription factor a gene by nuclear respiratory factors: a potential regulatory link between nuclear and mitochondrial gene expression in organelle biogenesis. Proc Natl Acad Sci U S A..

[15] Puigserver P, Wu Z, Park CW, Graves R, Wright M, Spiegelman BM (1998). A cold-inducible coactivator of nuclear receptors linked to adaptive thermogenesis. Cell..

[16] Kassam I, Qi T, Lloyd-Jones L, Holloway A, Jan Bonder M, Henders AK (2016). Evidence for mitochondrial genetic control of autosomal gene expression. Hum Mol Genet..

[17] Ballard JW, Youngson NA. Review: can diet influence the selective advantage of mitochondrial DNA haplotypes? Biosci Rep. 2015;35(6). 10.1042/BSR20150232 PMC4708006 26543031

[18] Salin K, Auer SK, Rey B, Selman C, Metcalfe NB (2015). Variation in the link between oxygen consumption and ATP production, and its relevance for animal performance. Proc Biol Sci..

[19] Migliaccio V, Sica R, Di Gregorio I, Putti R, Lionetti L. High-Fish Oil and High-Lard Diets Differently Affect Testicular Antioxidant Defense and Mitochondrial Fusion/Fission Balance in Male Wistar Rats: Potential Protective Effect of omega3 Polyunsaturated Fatty Acids Targeting Mitochondria Dynamics. Int J Mol Sci. 2019;20(12). 10.3390/ijms20123110 PMC6627479 31242698

[20] Lionetti L, Mollica MP, Donizzetti I, Gifuni G, Sica R, Pignalosa A (2014). High-lard and high-fish-oil diets differ in their effects on function and dynamic behaviour of rat hepatic mitochondria. PLoS One..

[21] Park H, He A, Tan M, Johnson JM, Dean JM, Pietka TA (2019). Peroxisome-derived lipids regulate adipose thermogenesis by mediating cold-induced mitochondrial fission. J Clin Invest..

[22] Cioffi F, Senese R, Lasala P, Ziello A, Mazzoli A, Crescenzo R, et al. Fructose-Rich Diet Affects Mitochondrial DNA Damage and Repair in Rats. Nutrients. 2017;9(4). 10.3390/nu9040323 PMC5409662 28338610

[23] Aw WC, Towarnicki SG, Melvin RG, Youngson NA, Garvin MR, Hu Y (2018). Genotype to phenotype: diet-by-mitochondrial DNA haplotype interactions drive metabolic flexibility and organismal fitness. PLoS Genet..

[24] Waerp HKL, Waters SM, McCabe MS, Cormican P, Salte R (2018). RNA-seq analysis of bovine adipose tissue in heifers fed diets differing in energy and protein content. PLoS One..

[25] Novais FJ, Pires PRL, Alexandre PA, Dromms RA, Iglesias AH, Ferraz JBS (2019). Identification of a metabolomic signature associated with feed efficiency in beef cattle. BMC Genomics..

[26] Van Elswyk ME, McNeill SH (2014). Impact of grass/forage feeding versus grain finishing on beef nutrients and sensory quality: the U.S. experience. Meat Sci..

[27] McAfee AJ, McSorley EM, Cuskelly GJ, Fearon AM, Moss BW, Beattie JA (2011). Red meat from animals offered a grass diet increases plasma and platelet n-3 PUFA in healthy consumers. Br J Nutr..

[28] Daley CA, Abbott A, Doyle PS, Nader GA, Larson S (2010). A review of fatty acid profiles and antioxidant content in grass-fed and grain-fed beef. Nutr J..

[29] Carrillo JA, He Y, Li Y, Liu J, Erdman RA, Sonstegard TS (2016). Integrated metabolomic and transcriptome analyses reveal finishing forage affects metabolic pathways related to beef quality and animal welfare. Sci Rep..

[30] Li Y, Carrillo JA, Ding Y, He Y, Zhao C, Liu J (2015). Transcriptomic profiling of spleen in grass-fed and grain-fed Angus cattle. PLoS One..

[31] Li Y, Carrillo JA, Ding Y, He Y, Zhao C, Zan L (2015). Ruminal Transcriptomic analysis of grass-fed and grain-fed Angus beef cattle. PLoS One..

[32] Ekstrand MI, Falkenberg M, Rantanen A, Park CB, Gaspari M, Hultenby K (2004). Mitochondrial transcription factor a regulates mtDNA copy number in mammals. Hum Mol Genet..

[33] Clemente P, Pajak A, Laine I, Wibom R, Wedell A, Freyer C (2015). SUV3 helicase is required for correct processing of mitochondrial transcripts. Nucleic Acids Res..

[34] Reverter A, Okimoto R, Sapp R, Bottje WG, Hawken R, Hudson NJ (2017). Chicken muscle mitochondrial content appears co-ordinately regulated and is associated with performance phenotypes. Biol Open..

[35] Pertea M, Kim D, Pertea GM, Leek JT, Salzberg SL (2016). Transcript-level expression analysis of RNA-seq experiments with HISAT, StringTie and Ballgown. Nat Protoc.

[36] Shi L, Westerhuis JA, Rosen J, Landberg R, Brunius C (2019). Variable selection and validation in multivariate modelling. Bioinformatics..

[37] Chong J, Wishart DS, Xia J (2019). Using MetaboAnalyst 4.0 for Comprehensive and Integrative Metabolomics Data Analysis. Curr Protoc Bioinformatics.

[38] Calvo SE, Clauser KR, Mootha VK (2016). MitoCarta2.0: an updated inventory of mammalian mitochondrial proteins. Nucleic Acids Res..

[39] Sun HZ, Wang DM, Liu HY, Liu JX (2018). Metabolomics integrated with Transcriptomics reveals a subtle liver metabolic risk in dairy cows fed different crop by-products. Proteomics..

[40] Humer E, Kroger I, Neubauer V, Reisinger N, Zebeli Q (2019). Supplementation of a clay mineral-based product modulates plasma metabolomic profile and liver enzymes in cattle fed grain-rich diets. Animal..

[41] Stump CS, Short KR, Bigelow ML, Schimke JM, Nair KS (2003). Effect of insulin on human skeletal muscle mitochondrial ATP production, protein synthesis, and mRNA transcripts. Proc Natl Acad Sci U S A..

[42] Fernandez-Vizarra E, Enriquez JA, Perez-Martos A, Montoya J, Fernandez-Silva P (2011). Tissue-specific differences in mitochondrial activity and biogenesis. Mitochondrion..

[43] Zickermann V, Kerscher S, Zwicker K, Tocilescu MA, Radermacher M, Brandt U (2009). Architecture of complex I and its implications for electron transfer and proton pumping. Biochim Biophys Acta..

[44] Brand MD, Nicholls DG (2011). Assessing mitochondrial dysfunction in cells. Biochem J..

[45] Reznik E, Wang Q, La K, Schultz N, Sander C. Mitochondrial respiratory gene expression is suppressed in many cancers. Elife. 2017;6. 10.7554/eLife.21592 PMC5243113 28099114

[46] Eslamieh M, Williford A, Betran E (2017). Few nuclear-encoded mitochondrial gene duplicates contribute to male Germline-specific functions in humans. Genome Biol Evol..

[47] Kadenbach B, Barth J, Akgun R, Freund R, Linder D, Possekel S (1995). Regulation of mitochondrial energy generation in health and disease. Biochim Biophys Acta..

[48] Taanman JW, Hall RE, Tang C, Marusich MF, Kennaway NG, Capaldi RA (1993). Tissue distribution of cytochrome c oxidase isoforms in mammals. Characterization with monoclonal and polyclonal antibodies. Biochim Biophys Acta..

[49] Radford NB, Wan B, Richman A, Szczepaniak LS, Li JL, Li K (2002). Cardiac dysfunction in mice lacking cytochrome-c oxidase subunit VIaH. Am J Physiol Heart Circ Physiol..

[50] Quintens R, Singh S, Lemaire K, De Bock K, Granvik M, Schraenen A (2013). Mice deficient in the respiratory chain gene Cox6a2 are protected against high-fat diet-induced obesity and insulin resistance. PLoS One..

[51] Hudson G, Chinnery PF (2006). Mitochondrial DNA polymerase-gamma and human disease. Hum Mol Genet.

[52] Garcia-Gomez S, Reyes A, Martinez-Jimenez MI, Chocron ES, Mouron S, Terrados G (2013). PrimPol, an archaic primase/polymerase operating in human cells. Mol Cell..

[53] Varma H, Faust PL, Iglesias AD, Lagana SM, Wou K, Hirano M (2016). Whole exome sequencing identifies a homozygous POLG2 missense variant in an infant with fulminant hepatic failure and mitochondrial DNA depletion. Eur J Med Genet..

[54] Porter GA Jr, Beutner G. Cyclophilin D, Somehow a Master Regulator of Mitochondrial Function. Biomolecules. 2018;8(4). 10.3390/biom8040176 PMC6316178 30558250

[55] Karch J, Bround MJ, Khalil H, Sargent MA, Latchman N, Terada N (2019). Inhibition of mitochondrial permeability transition by deletion of the ANT family and CypD. Sci Adv.

[56] Etzler JC, Bollo M, Holstein D, Deng JJ, Perez V, Lin DT (2017). Cyclophilin D over-expression increases mitochondrial complex III activity and accelerates supercomplex formation. Arch Biochem Biophys..

[57] Chui A, Murthi P, Gunatillake T, Brennecke SP, Ignjatovic V, Monagle PT (2014). Altered decorin leads to disrupted endothelial cell function: a possible mechanism in the pathogenesis of fetal growth restriction?. Placenta..

[58] Seidler DG (2012). The galactosaminoglycan-containing decorin and its impact on diseases. Curr Opin Struct Biol..

[59] Neill T, Torres A, Buraschi S, Owens RT, Hoek JB, Baffa R (2014). Decorin induces mitophagy in breast carcinoma cells via peroxisome proliferator-activated receptor gamma coactivator-1alpha (PGC-1alpha) and mitostatin. J Biol Chem..

[60] Rak M, Rustin P (2014). Supernumerary subunits NDUFA3, NDUFA5 and NDUFA12 are required for the formation of the extramembrane arm of human mitochondrial complex I. FEBS Lett..

[61] Ostergaard E, Rodenburg RJ, van den Brand M, Thomsen LL, Duno M, Batbayli M (2011). Respiratory chain complex I deficiency due to NDUFA12 mutations as a new cause of Leigh syndrome. J Med Genet..

[62] Patel D, Witt SN (2017). Ethanolamine and Phosphatidylethanolamine: Partners in Health and Disease. Oxidative Med Cell Longev..

[63] Broeks MH, Shamseldin HE, Alhashem A, Hashem M, Abdulwahab F, Alshedi T, et al. MDH1 deficiency is a metabolic disorder of the malate-aspartate shuttle associated with early onset severe encephalopathy. Hum Genet. 2019. 10.1007/s00439-019-02063-z 31538237

[64] Madeo F, Eisenberg T, Pietrocola F, Kroemer G. Spermidine in health and disease. Science. 2018;359(6374). 10.1126/science.aan2788 29371440

[65] Zhang H, Li T (2017). Effects of spermidine and ATP on stabilities of chromatosomes and histone H1-depleted chromatosomes. Bioorg Med Chem Lett..

[66] Lozoya OA, Martinez-Reyes I, Wang T, Grenet D, Bushel P, Li J (2018). Mitochondrial nicotinamide adenine dinucleotide reduced (NADH) oxidation links the tricarboxylic acid (TCA) cycle with methionine metabolism and nuclear DNA methylation. PLoS Biol..

