# Correction to: Diet induced the change of mtDNA copy number and metabolism in Angus cattle

**DOI:** 10.1186/s40104-020-00504-8

**Published:** 2020-08-25

**Authors:** Ying Bai, José A. Carrillo, Yaokun Li, Yanghua He, Jiuzhou Song

**Affiliations:** 1 College of Life Sciences and Food Engineering, Hebei University of Engineering, Handan, 056038 China; 2 Department of Animal & Avian Sciences, University of Maryland, College Park, MD 20742 USA; 3 Council on Dairy Cattle Breeding, Bowie, MD 20716 USA; 4 Human Nutrition, Food and Animal Sciences, University of Hawaii at Manoa, Honolulu, HI 96822 USA

**Correction to: J Animal Sci Biotechnol 11, 84 (2020)**

**https://doi.org/10.1186/s40104-020-00482-x**

In the original publication of this article [1], the author indicates that one of the GEO accession number is incorrect in the Availability of data and materials section.

The “https://www.ncbi.nlm.nih.gov/geo/query/acc.cgi?acc=GSE145376” should be “https://www.ncbi.nlm.nih.gov/geo/query/acc.cgi?acc=GSE145375”.

The original publication has been corrected.
