# Supplementary figures and images for: Diet induced the change of mtDNA copy number and metabolism in Angus cattle
Source: J Anim Sci Biotechnol. 2020 Jul 21;11:84. doi: 10.1186/s40104-020-00482-x (PMC7372754; doi:10.1186/s40104-020-00482-x)

(A)

## Muscle

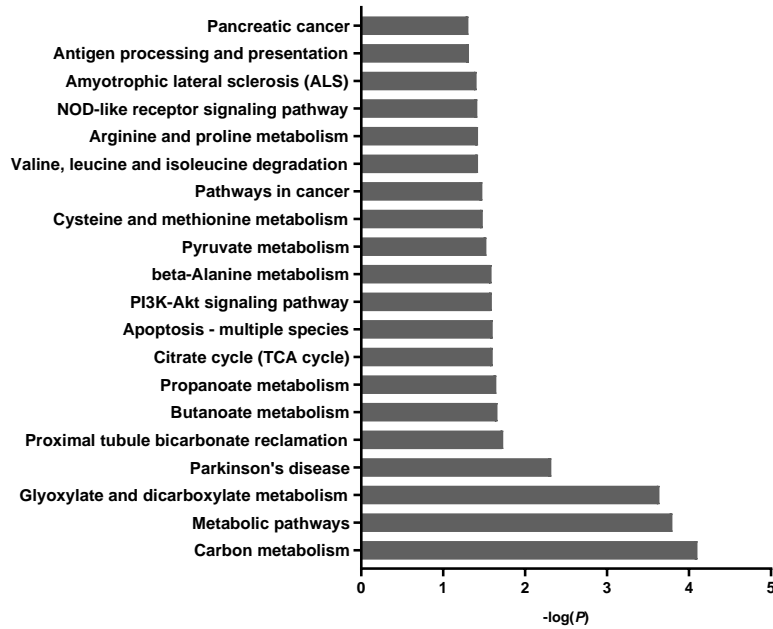

(B)

## Liver

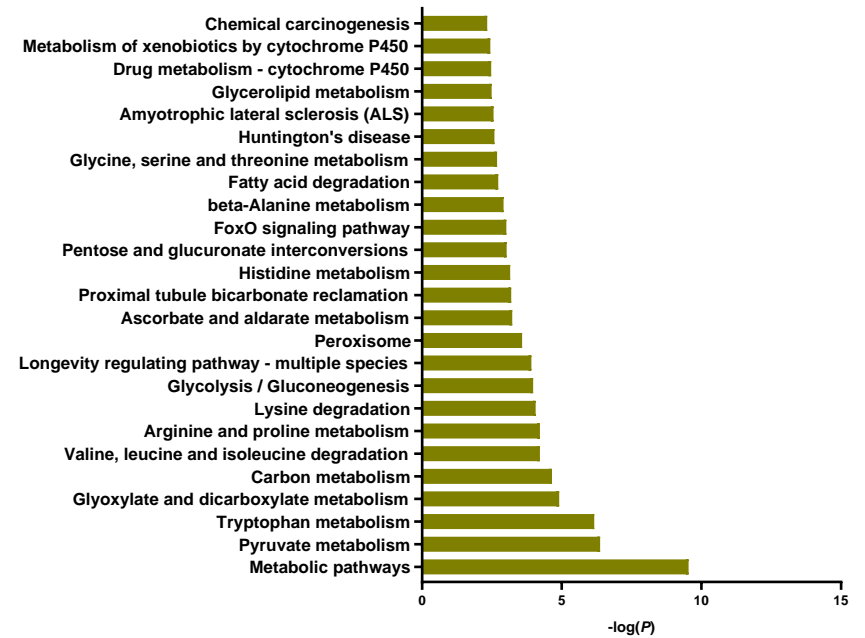

(C)

## Spleen

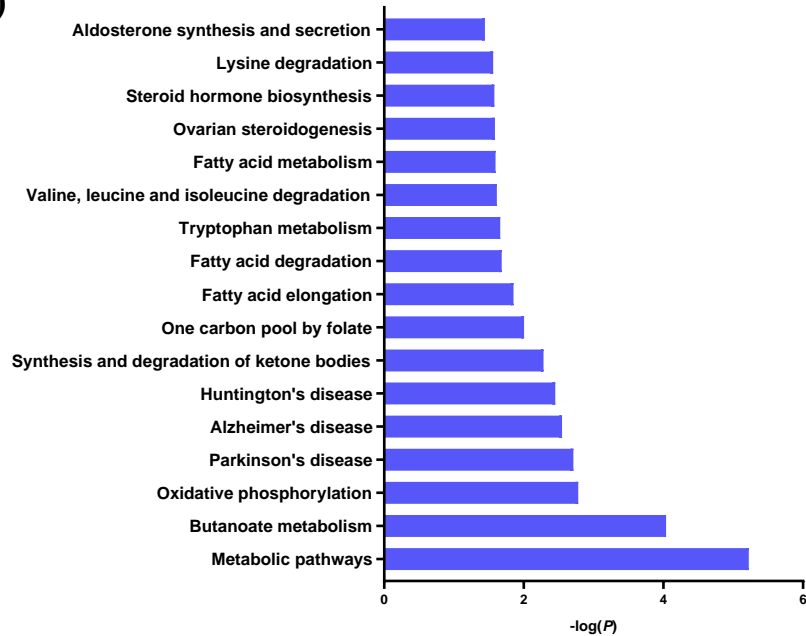

(D)

## Rumen

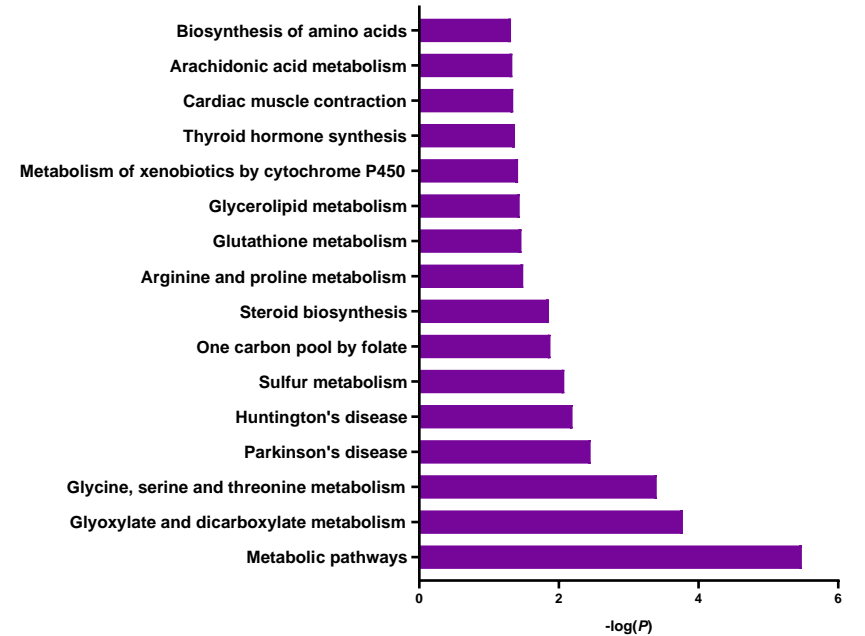

Supplement: Supplementary file 2 — Additional file 2: Figure S1. KEGG pathways differentially expressed between grass-fed and grain-fed Angus cattle. (A) muscle, (B) liver, (C) spleen, (D) rumen. [file 40104_2020_482_MOESM2_ESM.pdf]
